# Theoretical requirements for broadband perfect absorption of acoustic waves by ultra-thin elastic meta-films

**DOI:** 10.1038/srep12139

**Published:** 2015-07-17

**Authors:** Yuetao Duan, Jie Luo, Guanghao Wang, Zhi Hong Hang, Bo Hou, Jensen Li, Ping Sheng, Yun Lai

**Affiliations:** 1 College of Physics, Optoelectronics and Energy & Collaborative Innovation Center of Suzhou Nano Science and Technology, Soochow University, Suzhou 215006, China; 2 Department of Physics, Hong Kong University of Science and Technology, Clear Water Bay, Hong Kong; 3 School of Physics and Astronomy, University of Birmingham, Birmingham B15 2TT, UK

## Abstract

We derive and numerically demonstrate that perfect absorption of elastic waves can be achieved in two types of ultra-thin elastic meta-films: one requires a large value of almost pure imaginary effective mass density and a free space boundary, while the other requires a small value of almost pure imaginary effective modulus and a hard wall boundary. When the pure imaginary density or modulus exhibits certain frequency dispersions, the perfect absorption effect becomes broadband, even in the low frequency regime. Through a model analysis, we find that such almost pure imaginary effective mass density with required dispersion for perfect absorption can be achieved by elastic metamaterials with large damping. Our work provides a feasible approach to realize broadband perfect absorption of elastic waves in ultra-thin films.

Sound absorption within deep sub-wavelength space, especially in the low frequency regime, is an interesting and challenging issue. Traditional methods such as porous materials, micro-perforated plates, and sound absorption wedges show severe limitation in this respect. Recently, the rapid development of acoustic metamaterials^1^,^2^,^3^,^4^,^5^,^6^,^7^,^8^,^9^,^10^,^11^,^12^,^13^,^14^,^15^,^16^,^17^,^18^,^19^,^20^,^21^,^22^,^23^,^24^,^25^,^26^,^27^,^28^,^29^,^30^,^31^,^32^,^33^,^34^,^35^,^36^,^37^,^38^,^39^, i.e. artificial acoustic materials with almost arbitrary mass density and modulus, has provided new possibilities to achieve sound absorption in unprecedented ways. Bestowed with the unusual parameters, acoustic and elastic metamaterials exhibit strong abilities to control acoustic and elastic waves, giving rise to novel phenomena like low frequency sound blocking^1^,^2^, negative refraction and lensing^7^,^8^,^9^,^10^,^11^,^12^,^13^,^14^,^15^,^16^,^17^, acoustic cloaking^19^,^20^,^21^,^22^,^23^,^24^,^25^, etc. Very recently, acoustic metamaterials have been applied to enhance the absorption of acoustic wave energy. A “dark” acoustic metamaterial composed of a resonant membrane structure has been realized to absorb sound waves with wavelengths much larger than the thickness of the stricture^26^. Further studies revealed that impedance matching is the mechanism leading to perfect absorption in such ultra-thin structures, which may be regarded as acoustic meta-surfaces, and demonstrated a high efficiency of converting acoustic energy into electricity^27^. Another novel design is a bubble metascreen which has been demonstrated to exhibit broadband absorption of sound in water^28^. In the picture of coherent perfect absorption, perfect absorption of acoustic waves has been theoretically proved^29^,^30^. Other designs to enhance acoustic absorption include porous lamella-crystals^31^, metamaterial absorbers^32^,^33^,^34^,^35^, acoustic black holes^36^,^37^,^38^,^39^,^40^,^41^, etc. However, despite of so many designs, the theoretical requirements for broadband perfect absorption with a thin film has not been clearly addressed yet. In addition, the absorption of elastic waves is also an important issue with wide applications in seismic waves, damping systems, etc.

In this work, we analyze the mechanism and theoretical requirements to achieve broadband perfect absorption for elastic waves by using an ultra-thin elastic meta-film. Based on transfer matrix theory, we find out two types of ultra-thin films with such possibility. One requires one a large value of almost pure imaginary effective mass density and a free space boundary, while the other requires a small value of almost pure imaginary effective modulus and a hard wall boundary. In the former case, the displacement is almost a constant across the ultra-thin film, while the stress tends to zero abruptly. In the latter case, the situation is the opposite with the stress being almost a constant and the displacement tending to zero abruptly. We further show that when the almost pure imaginary effective mass density is inversely proportional to frequency, or the almost pure imaginary effective modulus is proportional to frequency, the perfect absorption effect becomes broadband. Through a simple model analysis, we demonstrate that elastic metamaterials with large damping can be designed to realize such effective media with almost pure imaginary parameters with the required frequency dispersions in certain frequency regimes, therefore providing a feasible approach for broadband absorption of elastic waves.

We consider elastic waves with angular frequency *ω* propagating along the *z* direction, which are incident on an ultra-thin film embedded in a background. Here, we assume the background and the film are both composed of isotropic solids. Therefore, when the incidence is in the normal direction, the transverse and longitudinal waves are uncoupled. For simplicity, in the following we only consider longitudinal waves of normal incidence. Similar conclusions can be obtained for transverse waves.

If we define *r*^*u*^ (*r*^*τ*^) and *t*^*u*^ (*t*^*τ*^) as the transmission and reflection coefficients through the ultra-thin elastic meta-film, with respect to the displacement (stress), respectively. Then, the transmission and reflection may be classified into four cases. In case 1, both the stress *τ* and displacement *u* are almost constants across the film. In this case, the transmission is almost unity and the reflection is almost zero. And there is no absorption. In case 2, the displacement *u* is almost a constant across the film, while the stress *τ* varies abruptly across the film. In this case, we have


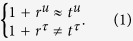


In case 3, the stress *τ* is almost a constant across the film, while the displacement *u* varies abruptly across the film. In this case, we have
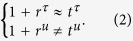


In case 4, both the stress *τ* and displacement *u* vary abruptly across the film. In this case, we have 1 + *r*^*τ*^ ≠ *t*^*τ*^ and 1 + *r*^u^ ≠ *t*^*u*^. Actually, case 4 indicates that the wavelength inside the film is generally comparable to the thickness of the film, therefore resulting in a non-negligible phase change across the film. In this work, we will focus on cases 2 and 3. As we shall show later, such cases exhibit interesting possibilities of achieving broadband perfect absorption, while case 4, though also capable of achieving perfect absorption, is usually limited to a narrow bandwidth due to the resonance effect.

In the following, we first analytically derive the conditions for the elastic waves to be perfectly absorbed based on a transfer matrix approach^42^,^43^. We denote the mass density, Lamé’s first and second parameters of the ultra-thin film (background medium) to be *ρ*(*ρ*_0_), *λ*(*λ*_0_) and *μ*(*μ*_0_), respectively.

We define a transfer matrix as,


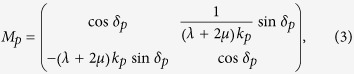


which establishes the relationship between 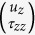 at *z* = 0 and *z* = *d*, in the form of


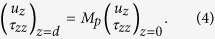


Here *k*_*p*_ is the wave number of longitudinal wave, *d* is the thickness of the ultra-thin film. And *δ*_*p*_ (=*k*_*p*_*d*) is the phase change across the film. Thus, the reflection and transmission coefficients can be derived as,


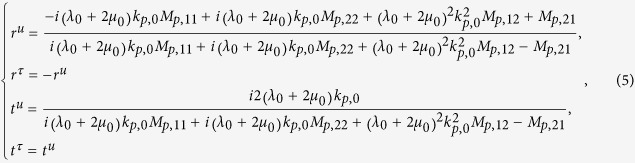


where *M*_*p,mn*_ is the element in *m*-th row and *n*-th column of the transfer matrix *M*_*p*_. *k*_*p*,0_ 
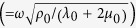
 is the wave number in the background medium. Here, we assume the phase change *δ*_*p*_ across the film is negligible, i.e.


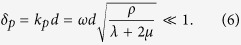


Thus, Eq. (5) can be simplified to


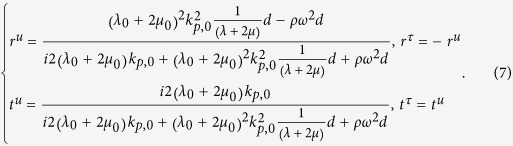


Now, we investigate the requirement of the parameters of the ultra-thin film and background in the cases 2 and 3 mentioned before. By substituting the reflection and transmission coefficients in Eq. (7) into Eqs (1) and (2), we obtain,


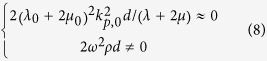


and


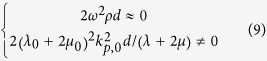


for cases 2 and 3, respectively. Since the ultra-thin film has *ωd *≪ 1, thus traditional solids have 2*ω*^2^*ρd* ≈ 0 and 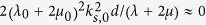. This indicates that an ultra-thin film made of traditional solids will exhibit almost constant displacement and stress across the film, leading to case 1 with no absorption. In order to realize cases 2 and 3 with absorption, from Eqs (8) and (9), we find that either the effective mass density of the film must be unusually large (case 2), or the effective modulus must be unusually small (case 3).

However, in cases 2 and 3, when the environment is symmetric (i.e., the media in the incident and transmitted regions are the same), from Eqs (1) and (2), we can easily find that the ultra-thin film has a maximal absorption rate of 50% with −*r*^*u*^ ≈ *t*^*u*^ ≈ 0.5 or −*r*^*τ*^ ≈ *t*^*τ*^ ≈ 0.5^44^.

In order to obtain perfect absorption, we must break the symmetry. Here, we employ a free space boundary or a hard wall boundary attached to the ultra-thin elastic meta-film, as illustrated in Fig. 1(a,b). Free space boundary enforces zero stress, while hard wall boundary enforces zero displacement. Therefore, free space boundary is associated with case 2, in which the displacement is almost a constant while the stress can decrease sharply to zero across the film. Hard wall boundary is associated with case 3, in which the stress is almost a constant while the displacement can decrease sharply to zero across the film. When the free space boundary is applied, the total reflection coefficient of the whole system is 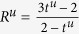 for case 2^44^. This implies that when the ultra-thin elastic meta-film satisfy *t*^*u*^ = *t*^*τ*^ = 2/3 and *r*^*u*^ = −*r*^*τ*^ = −1/3, perfect absorption can be achieved. On the other hand, when the hard wall boundary is applied, perfect absorption can be obtained when the ultra-thin elastic meta-film satisfies *t*^*u*^ = *t*^*τ*^ = 2/3 and *r*^*u*^ = −*r*^*τ*^ =1/3. Thus, from Eqs (7)–(9), 10, 11, the effective parameters of the ultra-thin film for perfect absorption can be derived as,


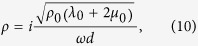


and


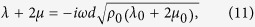


for case 2 with a free space boundary, and case 3 with a hard wall boundary, respectively.

From Eqs (10) and (11), we can see that when the imaginary effective mass density is inversely proportional to frequency, or the effective modulus is proportional to frequency, the perfect absorption condition can be satisfied in a broad range of frequencies, leading to broadband perfect absorption. As we shall prove later in model analysis, this type of dispersion is possible in damping elastic metamaterials. Two schematic graphs are shown in Fig. 1(a,b) to describe the physical picture of the two types of ultra-thin films, respectively.

Another interesting fact that is worth noting is that Eqs (10) only requires a particular value of imaginary effective mass density for the case of constant displacement, while the effective modulus of the film can vary within a relatively large range, as long as Eqs (1) and (8) are satisfied. Similarly, Eq. (11) only requires a particular value of imaginary effective modulus for the case of constant stress. Any effective mass density is fine as long as Eqs (2) and (9) are satisfied. This property allows us to focus on only one parameter instead of both of them, which greatly simplifies the design process.

Likewise, we can also derive the requirement of effective parameters for transverse waves as


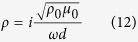


and


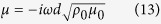


for the cases with almost constant displacement and shear stress, respectively.

From Eqs (10)–(13), 25, 28, 30, one may also find that generally the longitudinal and transverse waves cannot be perfectly absorbed simultaneously. In order to achieve perfect absorption, we require the effective mass density of the film to be anisotropic for case 2, or the effective modulus of the film to satisfy 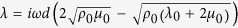 and 
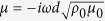
 for case 3.

In order to verify the analytical results, we perform numerical simulations based on finite element software, COMSOL Multi-physics, as shown in Fig. 2. The background material in the incident region is selected as epoxy with *λ*_0_ = 4.428 × 10^9^ Pa, *μ*_0_ = 1.590 × 10^9^ Pa and *ρ*_0_ = 1180 kg/m^3^. The incident waves from left are longitudinal waves with a working frequency of 500 Hz. Therefore, the wavelength in epoxy is about 5.08 m. In the simulation, we placed a ultra-thin elastic film with thickness of *d* = 0.1 m at the position about *z* = 5 m.

First, we consider the *u*-constant case. From Eq. (10), we obtain the required large imaginary mass density as *ρ* = *i*9538 kg/m^3^. Since there is no strict requirement on the modulus, we choose the same values of epoxy, i.e., *λ* = 4.428 × 10^9^ Pa and *μ* = 1.590 × 10^9^ Pa. In Fig. 2(a,b), we show the distributions of real part of displacement Re(*u*_*z*_) (color), normalized amplitudes of stress |*τ*_*zz*_|/|*τ*_*zz*,in_| (red solid lines) and displacement |*u*_*z*_|/|*u*_*z*,in_| (blue solid lines). *u*_*z*,in_ and *τ*_*zz*,in_ are, respectively, the normal displacement and normal stress of incident waves. Figure 2(a) demonstrates the transmission of longitudinal waves through the ultra-thin film embedded in epoxy, while Fig. 2(b) demonstrates the perfect absorption of wave energy when a free space boundary is attached to the right side of the film. From the normalized amplitudes in Fig. 2(a), it is observed that the transmission coefficient is about 2/3. The displacement is almost a constant across the film while the stress experiences an abrupt change due to large mass density. In Fig. 2(b), it is observed that when a free space boundary is attached, almost all the incident wave energy is absorbed. There are almost no reflected waves, as can be deducted from the nonexistence of variance in the normalized amplitudes in the incident region in Fig. 2(b).

Second, we consider the *τ*-constant case. From Eq. (11), we obtain the required small imaginary modulus as *λ* + 2*μ* = −*i*9.413 × 10^8^ Pa. In the simulations in Fig. 2(c,d), we choose *λ* = *μ* = −*i*3.138 × 10^8^ Pa. Since there is no strict requirement on the mass density, we choose the same value of epoxy as *ρ* = 1180 kg/m^3^. In Fig. 2(c,d), the distributions of Re(*u*_*z*_), |*τ*_*zz*_|/|*τ*_*zz*,in_|, and |*u*_*z*_|/|*u*_*z*,in_| are presented. Figure 2(c) shows the transmission of longitudinal waves through the ultra-thin film embedded in epoxy, while Fig. 2(d) demonstrates the perfect absorption of wave energy when a hard wall boundary is attached to the right side of the film. From the normalized amplitudes in Fig. 2(c), it is observed that the transmission coefficient is also about 2/3. However, unlike the case in Fig. 2(a), the stress is almost a constant across the film, while the displacement experiences an abrupt change due to the small modulus. In Fig. 2(d), it is observed that when a hard wall boundary is attached, almost all the incident wave energy is absorbed. There are almost no reflected waves, which can be deducted from the nonexistence of variance in the normalized amplitudes in the incident region in Fig. 2(d).

Moreover, we calculate the incident angle-dependent absorptance in Fig. 2(e) for the *u*-constant case (green lines) and *τ*-constant case (magenta lines) with the same material parameters as those in Fig. 2(b,d), respectively. It is seen that large absorption can be obtained in a wide range of incident angle. Therefore, the above numerical simulations coincide excellently with our analytical results. Although we only verify the longitudinal waves, the perfect absorption of transverse waves can be easily confirmed in a similar process.

Previously we have demonstrated that an ultra-thin film can with large imaginary mass density and a free space boundary, or with small imaginary modulus and a hard wall boundary can achieve perfect absorption of elastic waves. When the imaginary mass density is inversely proportional to frequency, or the imaginary modulus is proportional to frequency, such perfect absorption effect can be broadband. However, how to realize such imaginary parameters remains an unresolved issue. It is known that positive imaginary value of mass density and negative imaginary value of modulus correspond to absorption^29^. However, in most natural materials, the absorption is relatively small, rendering the parameters having relatively larger real parts than imaginary parts. In the following, we will propose a model of damping elastic metamaterials exhibiting effective mass density proportional to *i*/*ω*, therefore enabling the ability of broadband perfect absorption of elastic waves.

As illustrated by the inset of Fig. 3(a), we propose a simple one-dimensional mass-spring-mass model composed of a background mass *M*_I_ embedded with an inner mass *M*_II_, with large damping induced by the frictional losses between the two masses. We assume that the frictional losses are proportional to the velocity. Thus, according to Newton’s second law, we have,


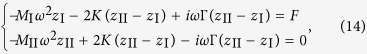


where *z*_I_ and *z*_II_ are the displacements of masses *M*_I_ and *M*_II_, respectively. *K* is the spring constant. Γ is damping constant, and *F* is an external force. We note that in Eq. (14), *z*_I_, *z*_II_ and *F* are assumed to vary time-harmonically with term *e*^−*iωt*^.

From the second equation in Eq. (14), we have 
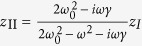
 with 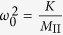 and 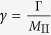. By eliminating *z*_II_ in the first equation in Eq. (14), we obtain 
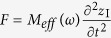
, where the effective mass is,


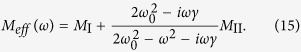


In particular, we find that if 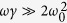, 
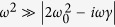
 and *γM*_II_/*ω* ≫*M*_I_, the effective mass of the model is,


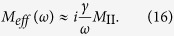


Equation (16) indicates that it is possible to obtain an effective mass density proportional to *i*/*ω* in the mass-spring-mass model, implying that such a model has the ability to achieve perfect absorption of elastic waves in a broad frequency regime.

For instance, we chose that parameters *M*_II_ = 1000*M*_I_ = 0.1 kg, *K* = 20 N/m and *γ* = 50 Hz. In Fig. 3(a), we plot the real part of effective mass Re(*M*_*eff*_) (blue solid lines) and its imaginary part Im(*M*_*eff*_) (red solid lines) with respect to the angular frequency *ω*, as obtained from Eq. (15). In addition, the imaginary part of effective mass calculated from Eq. (16) is also plotted as the green dashed lines in Fig. 3(a). It is found that Re(*M*_*eff*_) ≪ Im(*M*_*eff*_) and Im(*M*_*eff*_) ~ *iγM*_II_/*ω* for *ω* >100 Hz. From Eq. (15), it can be found that when *ω*_0 _≪ *γ* and *M*_II_ ≫ *M*_I_, Eq. (16) applies to a large frequency region between *γ* and *γM*_II_/*M*_I_. Therefore, if damping elastic metamaterials described by such a simple model can be realized, broadband perfect absorption can be achieved. We note that the working frequency is far away from the resonance frequency of the effective mass, which contributes to the broadband absorption effect.

There are other cases of perfect absorption in the above model, but they are all of narrow band. For instance, when *ω*_0_ ≫ *γ* and *M*_II_ ≫ *M*_I_, from Eq. (15), it can be found that at around frequency 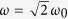, where the resonance occurs, the effective mass density is


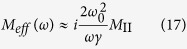


In Fig. 3(b), we plot the effective mass of a model with *M*_II_ = 1000*M*_I_ = 0.1 kg, *K* = 20 N/m and *γ* = 3 Hz. Blue and red solid lines denote the real and imaginary parts of effective mass *M*_*eff*_ obtained from Eq. (15). And green dashed lines denote the imaginary part of effective mass obtained from Eq. (17). We can see that the effective mass has a resonance at 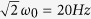, contributing to an abnormal dispersion of the real part and an enhancement of the imaginary part. Interestingly, in a narrow band falling in the abnormal dispersion region, the imaginary part of the effective mass dominates and satisfies Eq. (17). The absorption of such a model is a resonant absorption with most of energy transferring to heat through drastic friction. Compared with the model in Fig. 3(a), the model in Fig. 3(b) exhibits perfect absorption at a much narrower frequency regime.

In fact, if there is some way to fix the displacement of *M*_II_, i.e. *z*_II_, to be always zero, e.g. by connecting it with stiff materials to the earth, and if there is no spring connecting *M*_II_ and *M*_I_ (there is only frictional forces between them), then from the first equation in Eq. (14), we can obtain 
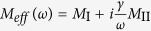
. In this case, when *ω* → 0, 
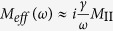
, indicating possibility of achieving broadband perfect absorption at extremely low frequencies.

The practical realization of such meta-film with natural materials requires careful design with the consideration of many minor effects such as nonlinearity as well as experimental verification. The difficulty mainly lies in finding natural materials of suitable mass density and realizing friction force that is proportional to velocity. Although we have developed our theory in the framework of elastic waves, it can also be applied to acoustic waves. Comparing with electromagnetic waves, for which we recently developed a theory to achieve perfect absorption with ultra-thin films^44^,^45^,^46^. The absorption of acoustic and elastic waves in ultra-thin films exhibits certain disadvantages and advantages. For electromagnetic waves, conductive films with almost pure imaginary permittivity 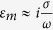 can be easily achieved with conductive materials like metals, where *σ* is the conductivity. The Drude plasma property of metal enables perfect absorption of low frequency electromagnetic waves. However, there are no natural materials for elastic and acoustic waves and damping elastic metamaterials have to be designed. Extremely low frequency broadband absorption is difficult as it either requires a fixed mass or a resonant system with large contrast masses and small *γ*. Especially, our analysis only applies to the linear regime with small displacements. However, there are also some advantages for elastic waves. For electromagnetic waves, the perfect magnetic conductor boundary is required, which is inherently narrow band^46^,^47^,^48^,^49^. However, for elastic waves, both free space and hard wall boundaries are naturally broadband, which makes the realization of broadband absorption of acoustic and elastic waves with an ultra-thin film easier than that of electromagnetic waves.

For conclusions, we have theoretically proved and numerically demonstrated the absorption of elastic waves in ultra-thin films with either imaginary large mass density and a free space boundary, or imaginary small modulus and a hard wall boundary. Broadband perfect absorption can be achieved when the frequency dispersions of the imaginary mass density or modulus can be inversely proportional to or proportional to the frequency in a certain frequency regime. We demonstrate that elastic metamaterials with large damping provides a feasible approach to realize the imaginary mass density with suitable dispersions for broadband absorption. Therefore, ultra-thin films composed of such metamaterials can in principle achieve broadband perfect absorption of elastic waves.

## Additional Information

**How to cite this article**: Duan, Y. *et al.* Theoretical requirements for broadband perfect absorption of acoustic waves by ultra-thin elastic meta-films. *Sci. Rep.* **5**, 12139; doi: 10.1038/srep12139 (2015).

## Figures and Tables

**Figure 1:**
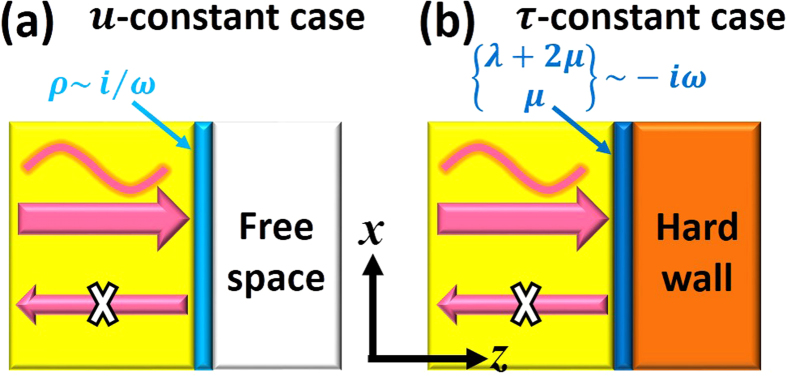
Schematic graph of perfect absorption by using an ultra-thin film (**a**) with free space for *u*-constant case, (**b**) with a hard wall for *τ*-constant case.

**Figure 2:**
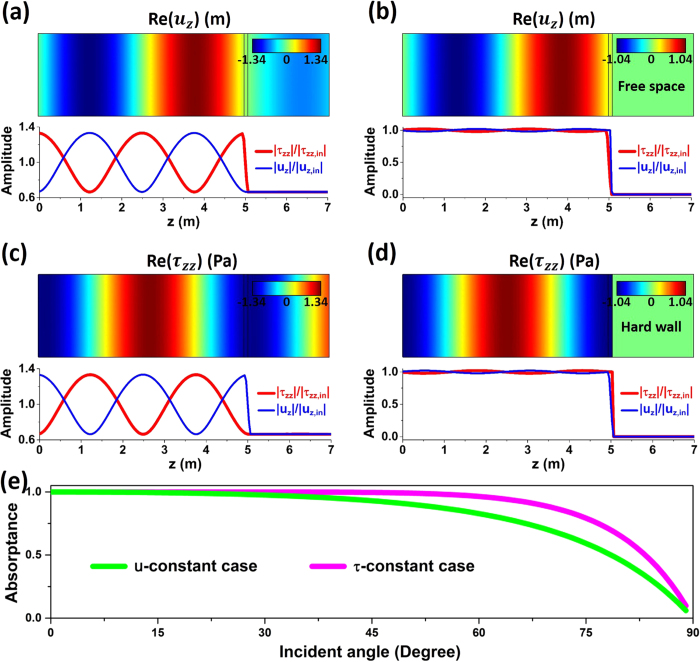
Simulations of perfect absorption. Snapshots of the real part of displacement Re(*u*_*z*_) (color), and normalized amplitudes of stress |*τ*_*zz*_|/|*τ*_*zz*,in_| (red solid lines) and displacement |*u*_*z*_|/|*u*_*z*,in_| (blue solid lines) for cases of (**a**) an ultra-thin film of imaginary mass density in epoxy, (**b**) with free space on the right side of the film. The parameters of the ultra-thin film are *λ* = 4.428 × 10^9^ Pa, *μ* = 1.590 × 10^9^ Pa, *ρ* = *i*9538 kg/m^3^ and *d* = 0.1 m. Snapshots of real part of stress Re(*τ*_*zz*_) (color), and normalized amplitudes of stress |*τ*_*zz*_|/|*τ*_*zz*,in_| (red solid lines) and displacement |*u*_*z*_|/|*u*_*z*,in_| (blue solid lines) for cases of (**c**) an ultra-thin film of imaginary bulk modulus in epoxy, (d) with hard wall on the right side of the film. The parameters of the ultra-thin film are *λ* = *μ* = −*i*3.138 × 10^8^ Pa, *ρ* = 1180 kg/m^3^ and *d* = 0.1 m. (**e**) Dependence of absorptance on the incident angle for the *u*-constant case (green lines) and *τ*-constant case (magenta lines) with the same material parameters as those in (**b**) and (**d**), respectively. The incident waves are longitudinal waves with working frequency 500 Hz.

**Figure 3:**
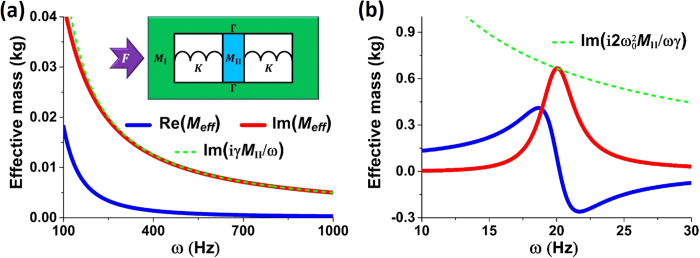
Effective mass of a one-dimensional lossy mass-spring-mass model as a function of the angular frequency *ω*. Effective mass of the model with (**a**) *M*_II_ = 1000*M*_I_ = 0.1 kg, *K* = 20 N/m, *γ* = 50 Hz, (**b**) *M*_II_ = 1000*M*_I_ = 0.1 kg, *K* = 20 N/m, *γ* = 3 Hz. Blue and red solid lines show the real and imaginary parts of effective mass *M*_*eff*_ obtained from Eq. (15). Green dashed lines in (**a**) and (**b**) show the imaginary part of effective mass obtained from Eq. (16) and Eq. (17), respectively. The inset in (**a**) is the schematic graph of the model.
